# Evolution of Microbial “Streamer” Growths in an Acidic, Metal-Contaminated Stream Draining an Abandoned Underground Copper Mine

**DOI:** 10.3390/life3010189

**Published:** 2013-02-07

**Authors:** Catherine M. Kay, Owen F. Rowe, Laura Rocchetti, Kris Coupland, Kevin B. Hallberg, D. Barrie Johnson

**Affiliations:** 1 College of Natural Sciences, Bangor University, Deiniol Road, Bangor, LL57 2UW, UK; E-Mail: c.kay@bangor.ac.uk; 2 Department of Ecology and Environmental Science, Umeå University, SE-901 87 Umeå, Sweden; E-Mail: owen.rowe@emg.umu.se; 3 Department of Life and Environmental Sciences, Università Politecnica delle Marche, Via Brecce Bianche, 60131 Ancona, Italy; E-mail: l.rocchetti@univpm.it; 4 College of Natural Sciences, Bangor University, Deiniol Road, Bangor, LL57 2UW, UK; E-Mail: kcoupland@hotmail.co.uk; 5 College of Natural Sciences, Bangor University, Deiniol Road, Bangor, LL57 2UW, UK; E-Mail: kevinhallberg@sky.com; 6 College of Natural Sciences, Bangor University, Deiniol Road, Bangor, LL57 2UW, UK

**Keywords:** *Acidithiobacillus*, acid mine drainage (AMD), acid streamers, *Actinobacteria*, *Ferrovum*, iron oxidation, microbial communities

## Abstract

A nine year study was carried out on the evolution of macroscopic “acid streamer” growths in acidic, metal-rich mine water from the point of construction of a new channel to drain an abandoned underground copper mine. The new channel became rapidly colonized by acidophilic bacteria: two species of autotrophic iron-oxidizers (*Acidithiobacillus ferrivorans* and “*Ferrovum myxofaciens*”) and a heterotrophic iron-oxidizer (a novel genus/species with the proposed name “*Acidithrix ferrooxidans*”). The same bacteria dominated the acid streamer communities for the entire nine year period, with the autotrophic species accounting for ~80% of the micro-organisms in the streamer growths (as determined by terminal restriction enzyme fragment length polymorphism (T-RFLP) analysis). Biodiversity of the acid streamers became somewhat greater in time, and included species of heterotrophic acidophiles that reduce ferric iron (*Acidiphilium, Acidobacterium, Acidocella* and gammaproteobacterium WJ2) and other autotrophic iron-oxidizers (*Acidithiobacillus ferrooxidans* and *Leptospirillum ferrooxidans*). The diversity of archaea in the acid streamers was far more limited; relatively few clones were obtained, all of which were very distantly related to known species of euryarchaeotes. Some differences were apparent between the acid streamer community and planktonic-phase bacteria. This study has provided unique insights into the evolution of an extremophilic microbial community, and identified several novel species of acidophilic prokaryotes.

## 1. Introduction

Acidophilic microorganisms are defined as those that grow optimally, or exclusively, in low pH environments, and have been sub-divided into moderate (pH optima 3–5) and extreme (pH optima < 3) acidophiles. The latter comprise a wide range of physiologically- and phylogenetically-diverse prokaryotes and a more limited number of eukaryotic microorganisms (chiefly micro-algae and protozoa [1]). Occasionally, individual cells of acidophilic microorganisms aggregate and form gelatinous macroscopic growths that have been referred to as “acid streamers” (filamentous growths in flowing waters), “acid mats” (thick and often dense growths), and “pipes” / “snotites” (pendulous growths attached to mine roofs or other subterranean features [1]). These have been found in subterranean and surface environments, frequently in and around abandoned mine sites [2]. The microbial composition of such growths is highly variable, and is determined to a great extent by the physico-chemical nature of the waters in which they develop. In the extremely acidic (pH 0–2) and warm (35–45 °C) streams and pools within the abandoned Richmond mine at Iron Mountain, California, iron-oxidizing bacteria (*Leptospirillum *spp.) and archaea (“*Ferroplasma acidarmanus*”) are often the dominant members of the acid streamer communities [3], whereas in higher pH (2–3) mine waters (e.g. at the Drei Konen und Ehrt pyrite mine in Germany, the Cae Coch pyrite mine in Wales) other iron-oxidizing bacteria (*Acidithiobacillus* spp. and “*Ferrovum myxofaciens*”) tend to dominate in sites rich in pyrite and other sulfide minerals [4]. The Frasassi cave complex in Italy provides an interesting contrast to pyrite-rich mines in that the main energy source for the acidophiles that form snotites is reduced sulfur rather than ferrous iron, and consequently the dominant prokaryotes are sulfur-oxidizers (*Acidithiobacillus thiooxidans*, with smaller numbers of *Sulfobacillus *and a bacterium related to *Acidimicrobium *[5]. Most data on the microbial compositions of acid streamers and snotites are from a single or relatively limited number of analyses of such growths whose “age” (in terms of how long they have been established) is unknown. Consequently, little is known about how these communities establish and evolve with time.

Mynydd Parys (Parys Mountain) is an abandoned copper mine located in the north-west corner of the island of Anglesey, north Wales [6]. In the 18th century it was the world's largest copper-producing mine. Although extractive mining ended in around 1880, copper was still recovered from the metal-rich waters draining the site until the 1950s. The two mines at Mynydd Parys (the larger Parys and smaller Mona mines) were operated initially as deep underground mines though towards the end of their working lives they were converted to opencast operations. In the 20th century, controlled flooding and drainage of the underground adits was used to recover copper using “*in situ”* bioleaching, though this was before the role of bacteria in this process had been recognized. When this practice was abandoned, the valves controlling the release of the mine water were closed, and consequently groundwater accumulated within the underground shafts and adits, forming a large pond in the opencast void of the Parys mine. Between ~1955 and 2003, the water level within the underground mine was controlled by overflow through an open adit (the Mona adit), and mine water flowed from this outlet eastwards forming a narrow stream (the Afon Goch [7]). Concern of the risk that this relatively high-elevation impounded acidic water body posed to the nearby low-level coastal town of Amlwch led to the partial dewatering of the mine in 2003. Between April and July, *ca*. 274,000 m^3^ of water was pumped from the mine into the Irish Sea, after which the retaining dam was destroyed, and the overflow water from the underground water body was constrained to flow northwards through a new channel which connected to an existing stream which had previously not received acid mine drainage (AMD) [8]. The diversion of mine water, draining Mynydd Parys into the new stream channel (the “Dyffryn Adda”), presented a unique opportunity to investigate how rapidly acidophilic microbial communities develop in a new drainage channel, and how the composition of these growths evolve with time. Here we report the results of a nine year study of the microbiology and geochemistry of the Dyffryn Adda from its inception as an AMD stream.

## 2. Results

### 2.1. Physico-Chemistry of Dyffryn Adda AMD

**Figure 1:**
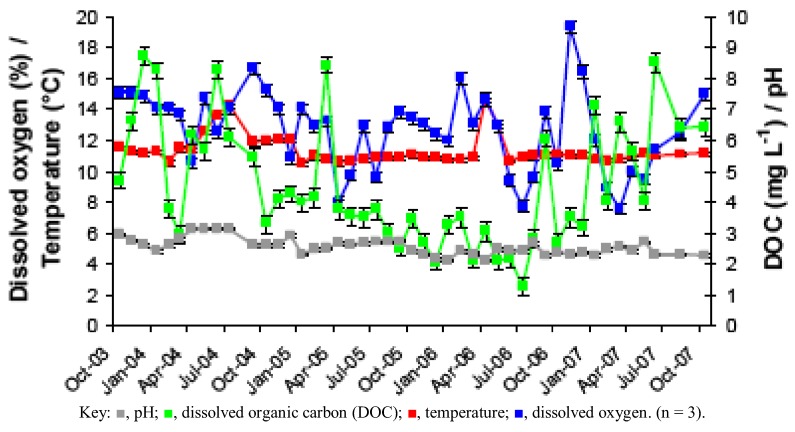
Changes in pH, temperature, dissolved oxygen and dissolved organic carbon (DOC) in acid mine drainage (AMD) at the Dyffryn Adda adit portal between October 2003 and November 2007.

**Figure 2:**
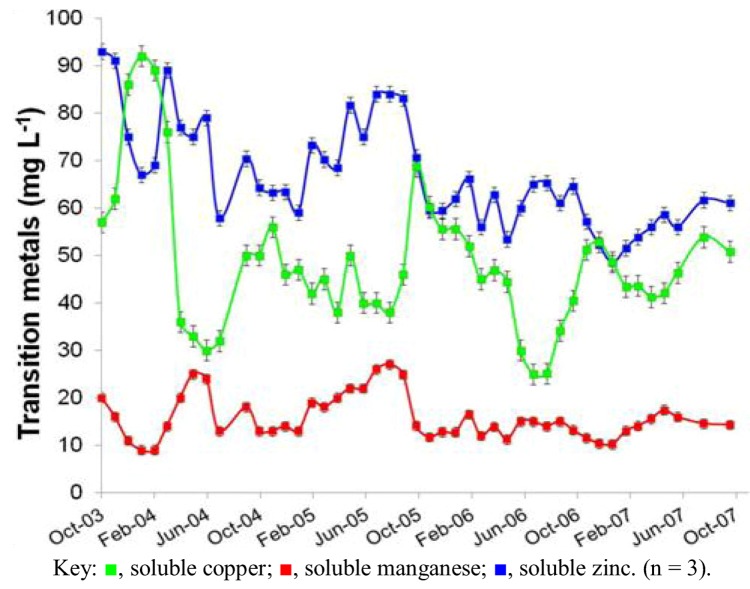
Changes in concentrations of transition metals in AMD at the Dyffryn Adda adit portal between October 2003 and November 2007.

**Figure 3:**
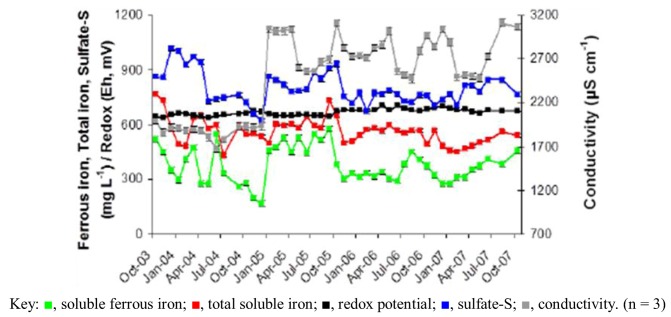
Changes in iron, sulfate-S, redox potential and conductivity in AMD at the Dyffryn Adda adit portal between October 2003 and November 2007.

**Figure 4:**
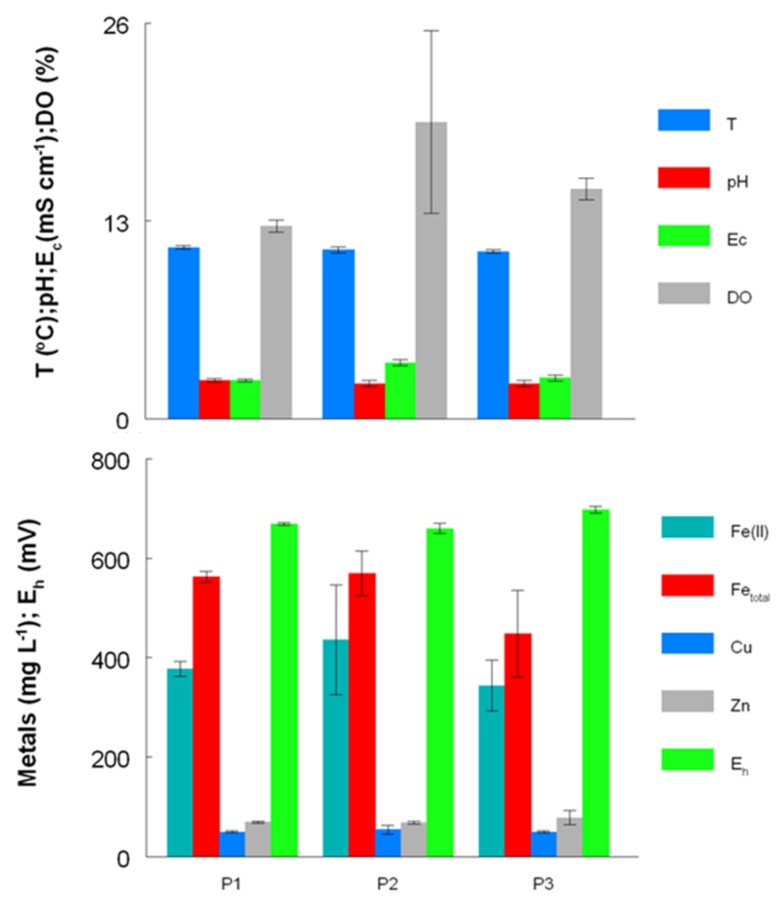
Concentrations of metals and other physico-chemical parameters (temperature (T), pH, conductivity (E_c_), redox potentials (E_h_) and dissolved oxygen concentrations (DO)) in AMD at the Dyffryn Adda adit portal between October 2003 and November 2007 (P1), November 2008 and August 2009 (P2) and February 2011 and August 2012 (P3).

Prior to overflow AMD from Mynydd Parys being diverted into the Dyffryn Adda, water flowing through the channel was mostly run-off and groundwater from the neighboring fields (used for agricultural grazing) was moderately acidic (pH ~5) and not contaminated with metals (data not shown). Analysis of mine water samples taken from the upper end of the Dyffryn Adda since October 2003 have shown only relatively minor changes in many of the parameters measured over the nine year period (Table 1 and Figure 1, Figure 2, Figure 3, Figure 4). The subterranean water body is sufficiently remote from the land surface not to be affected by seasonal temperature fluctuations, and water draining from it into the Dyffryn Adda has a near constant temperature of *ca*. 11 °C. Figure 1, Figure 2, Figure 3 show changes in the various physico-chemical parameters measured between October 2003 and November 2007 when the stream was monitored on a monthly basis. Concentrations of copper showed some variation between months one and 12, and mine water conductivity increased sharply at the start of 2005 compared to pre-2005 values. Concentrations of DOC also varied from time to time though values were not seasonally-related and did not appear to correlate with any other measured parameter. Comparison of data obtained during the first four years and those obtained later (grouped as three time periods: 2003–2007, 2008–2009 samples, and 2011–2012 samples) show that the AMD discharged in the Dyffryn Adda has continued to remain fairly constant in its physico-chemical composition during this time (Figure 4). Measurements of the parameters listed in Table 1 in the Dyffryn Adda from the adit portal at regular intervals to the point at which it joined with a second stream, showed that any changes were either not detectable or minor (data not shown). This was probably due to the relatively rapid flow rate of the stream, which was recorded by personnel of the Environment Agency (UK) to have a mean value of 10 L s^−1^.

**Table 1:** Mean physico-chemical data of mine water flowing from the Dyffryn Adda adit portal between October 2003 and November 2007. All concentrations shown are mg L^−1^, except where indicated (n=46).

Analyte	Mean value (standard error)
pH	2.53 (0.04)
Redox potential *E*_h_ (mV)	669 (2.6)
Conductivity (µS cm^−1^)	2506 (66)
Temperature (°C)	11.3 (0.1)
Oxygen (%)	12.7 (0.4)
Sulfate-S	800 (13)
Fe^2+^	378 (14)
Fe_total_	563 (11)
Zn	67 (1.6)
Cu	49 (2.2)
Mn	15.8(0.7)
Al	2.15(0.16)
DOC*	4.50(0.29)

*dissolved organic carbon.

### 2.2. Growth of Acid Streamers within the Dyffryn Adda

**Figure 5:**
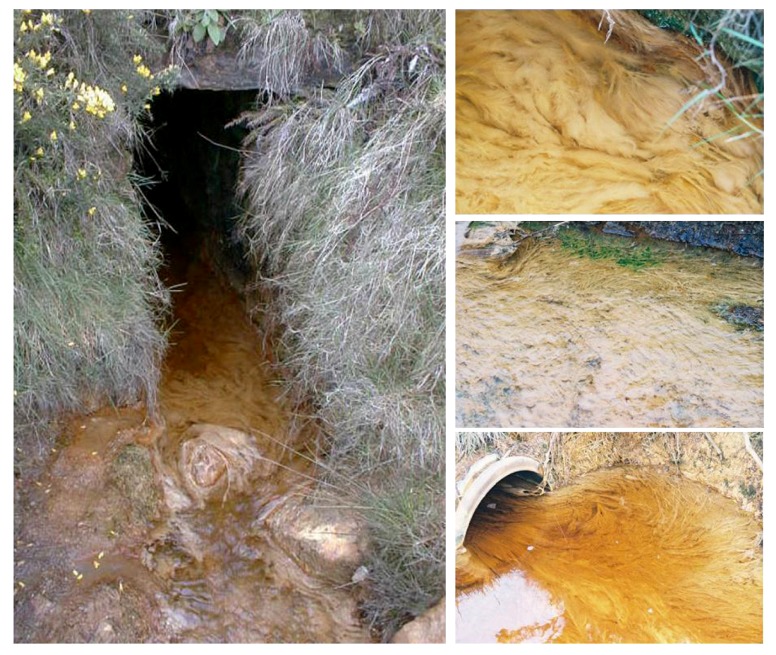
Acid streamer growths within the Dyffryn Adda, two years after acid mine drainage water from Mynydd Parys was diverted into this stream: (left) the screened portal at the upper end of the drainage channel; (right top) close up image of acid streamer growths close to the portal; (right middle) 15 m downstream of the portal, with *Euglena*-colonised surface streamers close to the stream bank; (right bottom) 90 m downstream of the portal, just before the Dyffryn Adda merges with a second stream.

Macroscopic gelatinous microbial growths were found in the upper reaches of the Dyffryn Adda within three months of it receiving AMD from the abandoned copper mines. These rapidly colonized the entire length of the stream between the portal and its conjuncture with a second stream, forming long (up to 0.5 m) off-white filamentous growths that attached to rocks, branches and other structures in the stream. Occasionally, surfaces of some streamer growths were green-colored due to the presence of *Euglena mutabilis* [9] which had also previously been reported in the Afon Goch [7]. Figure 5 shows images of the acid streamers within the Dyffryn Adda taken during April 2005. The section of the stream from the adit portal to its juncture with a second stream has remained similarly fully colonized by acid streamer growths since that time. The conjoining stream has a circum-neutral pH, and the higher pH of the mixed waters causes ferric iron to hydrolyze and precipitate, enshrouding streamer growths downstream of the junction of the two streams.

### 2.3. Molecular Analysis of Acid Streamer Growths: Bacteria

**Figure 6:**
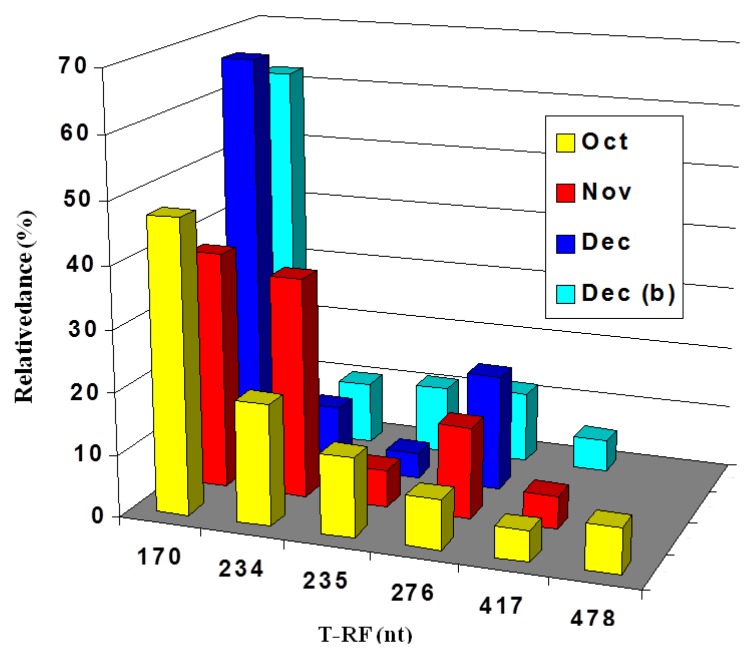
Terminal restriction enzyme fragment length polymorphism (T-RFLP) profiles of bacterial 16S rRNA genes (digested with AluI) of acid streamers sampled at the Dyffryn Adda adit portal, and 90 m downstream of the portal (Dec (b)). Sampling period: October–December 2003. The 170 nt fragment was attributed to “*Fv. myxofaciens*”, the 234 nt fragment to *At. ferrivoran*s and the 235 and 478 (a pseudo T-RF) nt fragments to the actinobacterium isolate Py-F3. The 276 nt fragment was also thought to be a pseudo-T-RF, and the 417nt fragment was notidentified.

Newly formed acid streamers sampled during 2003 showed that they were composed mainly of relatively few species of acidophilic bacteria (Figure 6). Streamers were sampled only at the Dyffryn Adda adit portal during October and November, while in December they were also sampled 90 m downstream of this point. terminal restriction enzyme fragment length polymorphism (T-RFLP) analysis indicated that the composition of the streamers was similar in all cases, with two species of autotrophic iron-oxidizing proteobacteria (“*Fv. myxofaciens*” and *Acidithiobacillus ferrivorans*) and a heterotrophic iron-oxidizing isolate (actinobacterium Py-F3) accounting for most of the biomass present.

**Figure 7:**
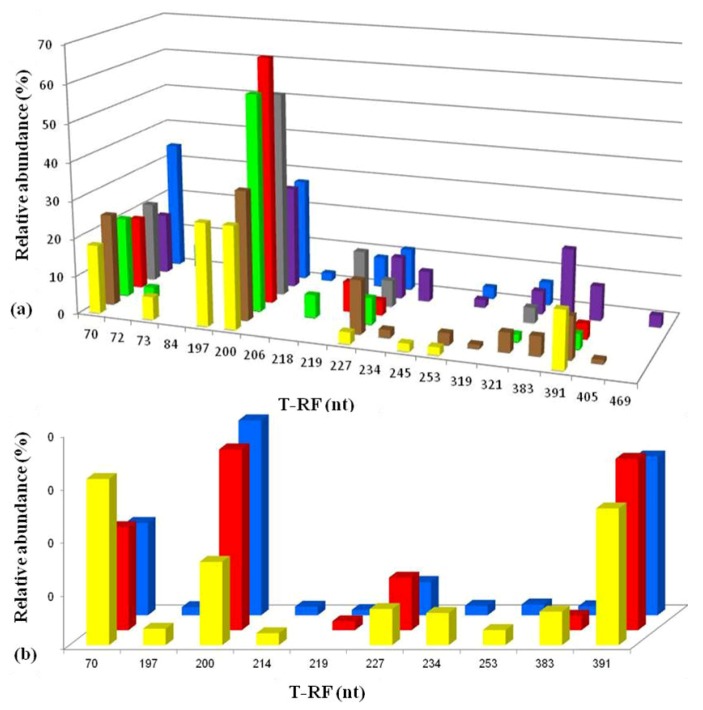
T-RFLP profiles of bacterial 16S rRNA genes (digested with HaeIII) of acid streamers from the Dyffryn Adda: (**a**) sampled during October 2007; (**b**) sampled during August 2012. Color code (bars): yellow, adit portal; brown, +15 m; green, +30 m; red, +45 m; charcoal, +60 m; purple, +75 m; blue, +90 m. The major peaks identified corresponded to *At. ferrivorans *(70 nt) and “*Fv. myxofaciens*” (200 nt), with smaller and more sporadic T-RFs corresponding to heterotrophic acidophiles (actinobacterium Py-F3 (227 nt), gammaproteobacterium WJ2 (197 nt) and *Acidobacterium* sp. Thars 1 (214 nt)), the iron-oxidizing chemoautotrophs *L. ferrooxidans *(206 nt) and *At. ferrooxidans* (253 nt), or plastid 16S rRNA (383, 391 nt). The 219 and 234 nt fragments found as minor T-RFs in two streamer samples were tentatively identified as corresponding to *Alicyclobacillus* clone DAAP3B4 or *Acidiphilium* spp. (both obligate heterotrophs). Other minor peaks were not identified.

On the next three sampling occasions (April 2005, October 2007 and November 2008) acid streamers were sampled at the upper portal and at 15 m intervals downstream (to 90 m) to examine the heterogeneity of the microbial communities on a spatial as well as on a temporal basis. In general, acid streamer samples from different points within the Dyffryn Adda showed very similar bacterial compositions, as shown in the T-RFLP profiles for October 2007 (Figure 7a; other data not shown). Occasionally, amplified plastid 16S rRNA genes (mostly from micro-algae) skewed the bacterial data somewhat, but when these were removed, a similar picture emerged to the nascent streamers. The chemoautotrophic iron-oxidizing autotrophic “*Fv. myxofaciens*” and *At. ferrivorans *were the dominant bacteria present, with other terminally labeled restriction fragment lengths (T-RFs) identified corresponding mostly to heterotrophic acidophiles (of which actinobacterium Py-F3 was usually the dominant bacterium). There was also a notable increase in the biodiversity of the streamer growths, with several additional species being either firmly (gammaproteobacterium WJ2, *At. ferrooxidans *and *Leptospirillum ferrooxidans*) or tentatively (*Acidiphilium* and *Alicyclobacillus* spp.) identified compared to those detected during the first months of sampling, though some of these (in contrast to *At. ferrivorans, *“*Fv. myxofaciens*” and actinobacterium Py-F3) were only detected sporadically.

Acid streamers in the Dyffryn Adda were analyzed further on six occasions between March 2009 and August 2012, and a representative T-RFLP profile of 16S rRNA genes amplified with bacteria-specific primers (from August 2012) is shown in Figure 7b. A general pattern of bacterial diversity similar to that observed between 2005 and 2008, though with occasional subtle differences, was found during this period. In addition, eukaryotic microorganisms (identified from T-RFs of digested plastid 16S rRNA genes) were occasionally present in relatively large amounts, particularly in the summer months, in streamers sampled between 2009 and 2012.

**Figure 8:**
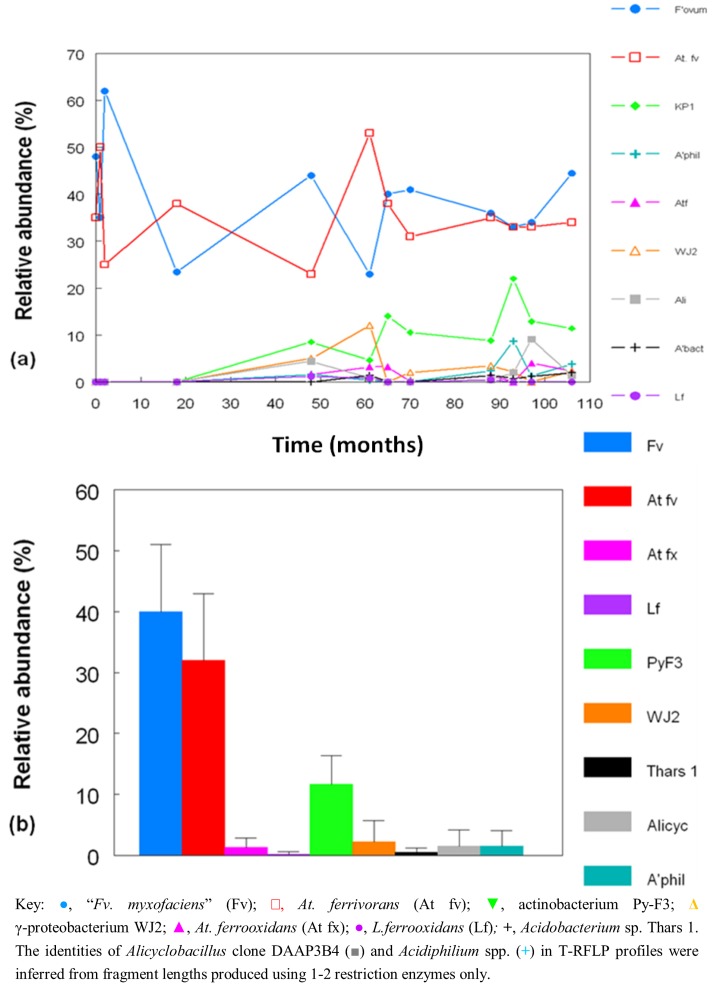
(**a**) Changes in the relative abundances of bacteria in acid streamers in Dyffryn Adda, determined by T-RFLP analysis of amplified 16S rRNA genes; (**b**) mean values and standard deviations of the relative abundance of different species of acidophilic bacteria in acid streams in the Dyffryn Adda, measured over a nine year sampling period. (n =42).

Figure 8a shows changes in the relative abundances of the bacteria identified in T-RFLP profiles over the nine year period covered by this study. Where multiple streamer samples were analyzed at any one time, average abundance data were calculated. Figure 8b shows the collated data for each of the acidophilic bacteria identified in the Dyffryn Adda streamers as mean values and standard deviations over the nine year sampling period. Data shown in Figure 8 have been corrected to take into account plastid genes which were also amplified by the bacterial primers used.

### 2.4. Bacterial Isolates and Cloned Genes

**Figure 9:**
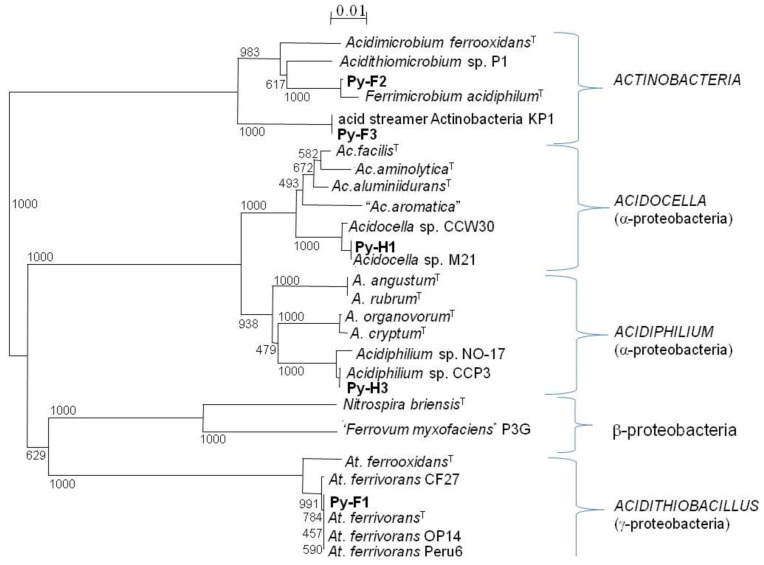
Unrooted tree showing the phylogenetic relationship of bacteria isolated from acid streamers in the Dyffryn Adda with known species of acidophiles, based on comparison of their 16S rRNA genes (720 nucelotides). The scale bar represents 1% nucleotide sequence divergence and numbers at the nodes are bootstrap values out of 1000 trials. Type strains are identified by a superscript T. The acid streamer isolates are indicated in bold text.

The dominant bacteria identified in T-RFLP profiles of acid streamers were isolated on solid media and their identities confirmed by analysis of their 16S rRNA genes (Table 2). These were “*Ferrovum myxofaciens*” (strain P3G), *Acidithiobacillus ferrivorans* (strain Py-F1), and a novel actinobacterium (strain Py-F3). Other isolates included a second heterotrophic iron-oxidizing actinobacterium (strain Py-F2) which was 99.5% identical (16S rRNA gene) to the type strain of *Ferrimicrobium acidiphilum*, and two strains of iron-reducing alphaproteobacteria, Py-H1 (97% identity to *Acidocella aluminidurans*) and Py-H3 (96% identity to *Acidiphilium rubrum*). The phylogenetic relationships of the Dyffryn Adda isolates to known species of acidophilic bacteria are shown in Figure 9.

### 2.5. Molecular Analysis of Acid Streamer Growths: Archaea

**Figure 10:**
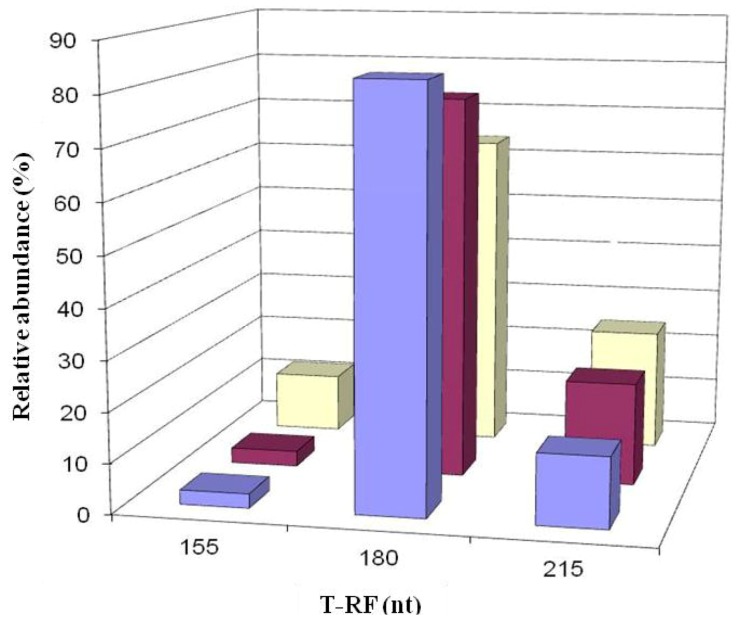
T-RFLP profiles of archaeal 16S rRNA genes (digested with HaeIII) of acid streamers from the Dyffryn Adda adit portal (violet bars), and at 45 m (magenta bars) and 90 m (cream bars) downstream of the portal, sampled during March 2009.

**Figure 11:**
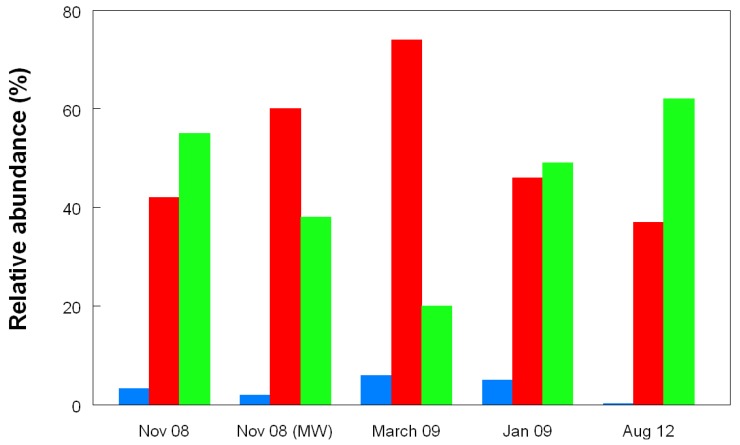
Changes in the relative abundances of archaea in acid streamers (and mine water in November 2008) in Dyffryn Adda, determined by T-RFLP analysis of amplified 16S rRNA genes. The red bars clone DAAP3A2 (180 nt), the green bars clones DAAP3A1, DAAP3A3 and DAAP3A6 (215 nt), and the blue bars represent unidentified archaea (155 nt). The gene fragment lengths referred to are from HaeIII digests and correspond to T-RFs shown in Figure 10.

No archaeal 16S rRNA genes were amplified from DNA extracted from acid streamers sampled in 2003. In 2005 and 2007, 16S rRNA archaeal genes were amplified though subsequent T-RFLP analysis of these was not successful. Archaeal diversity in the acid streamers was, however, elucidated in analyses carried out between 2008 and 2012. T-RFLP analysis indicated that the diversity of archaea was relatively limited, with two dominant T-RF peaks and one minor peak in T-RFLP profiles generated by HaeIII digests (Figure 10). Clones corresponding to two of these peaks were obtained in libraries and identified by comparing gene sequences to those in GenBank (Table 2). A single clone (DAAP3A2) corresponding to the 180 nt T-RF was obtained, whereas three clones, all distantly related to each other, had T-RFs of 215 nt length (HaeIII digests). All of the clones were most closely related (of known species) to euryarchaeotes of the order *Thermoplasmatales* (Table 2). Changes in the relative abundances of archaea corresponding to the three T-RFs are shown in Figure 11.

**Table 2:** Identities of microbial isolates and cloned genes obtained from acid streamers in the Dyffryn Adda.

Isolate/clone designation (GenBank Accession No.)	Closest relative (GenBank Accession No.)	Identity (%) (16S rRNA gene)	Reference
Bacterial isolates			
P3G (“*Ferrovum myxofaciens*”^T^) (HM044161)	“*Ferrovum myxofaciens*” PSTR (EF133508)	100	[10]
Py-H1 (KC208493)	*Acidocella *sp. strain M21(AY765998)	100	[10]
	*Acidocella aluminiidurans* (AB362219)	97.4	[11]
Py-H3 (KC208494^)^	*Acidiphilium *sp. CCP3(AY766000)	99.9	[10]
	*Acidiphilium *sp. NO-17 (AF376026)	99.5	[12]
	*Acidiphilium rubrum*^T^ (NR_025854)	95.8	[13]
Py-F1 (KC208495)	*Acidithiobacillus ferrivorans*^T^ (AF376020)	100	[14]
Py-F2 (KC208496)	*Ferrimicrobium acidiphilum*^T^ (NR_041798)	99.5	[15]
Py-F3 (“*Acidithrix ferrooxidans*”) (KC208497)	Heterotrophic iron-oxidizing bacterium KP1(AY765991)	100	[10]
	*Ferrimicrobium acidiphilum*^T^ (NR_041798)	92.4	[15]
Bacterial clones			
DAAP3B4 (KC208499)	*Alicyclobacillus* K23_bac (EF464642)	99.3	[16]
	*Alicyclobacillus* *ferrooxydans*^T^ (NR_044413)	90.0	[17]
PMC25 (KC208498)	*Actinobacterium *U2V-bac_a5 (JN982098)	95.9	[18]
	*Ferrimicrobium acidiphilum*^T^ (NR_041798)	94.8	[15]
	Isolate Py-F3	91.3	(this study)
Archaeal clones			
DAAP3A2 (KC208501)	Clone from sediment in an acidic pit lake (FJ228391)	99.1	(GenBank entry)
	*Methanomassiliicoccu* *luminyensis*^T^ (HQ896499)	83.5	[19]
DAAP3A1 (KC208500)	Clone from sediment in an acidic pit lake (FJ228392)	99.7	(GenBank entry)
	*Methanomassiliicoccus* *luminyensis*^T^ (HQ896499)	82.9	[19]
	Clone DAAP3A3	77.1	(this study)
	Clone DAAPA6	90.4	(this study)
DAAP3A3 (KC208502)	clone from coal-impacted forest wetland (AF523941)	98.5	[20]
	*Thermogymnomonas acidicola*^T^ (NR_041513)	90.6	[21]
	Clone DAAP3A6	76.8	(this study)
DAAP3A6 (KC2084503)	Clone from AMD stream (HE653789)	99.2	[22]
	*Methanomassiliicoccus luminyensis*^T^ (HQ896499)	80.6	[19]

### 2.6. Comparison of Acid Streamer and Planktonic Phase Prokaryotic Communities in the Dyffryn Adda

**Figure 12:**
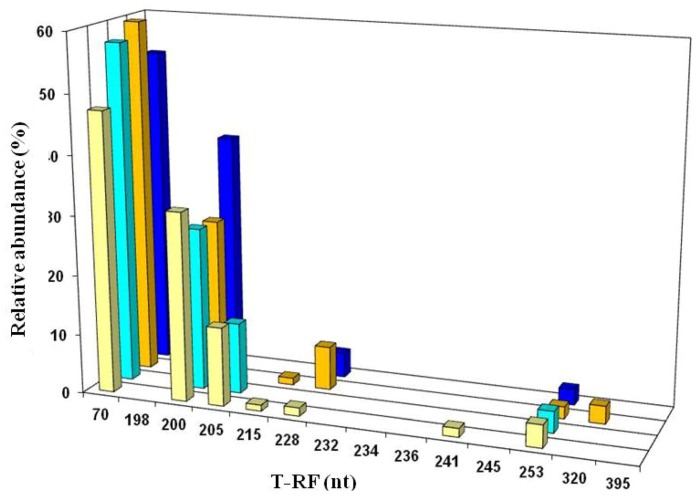
T-RFLP profiles of bacterial 16S rRNA genes (digested with HaeIII) of filtered mine water (cream and light blue bars) and acid streamers (orange and dark blue bars) from the Dyffryn Adda adit portal (cream and orange bars), and 90 m downstream of the portal (light and dark blue bars), sampled during November 2008. The major peaks identified corresponded to *At. ferrivorans *(70 nt), “*Fv. myxofaciens*” (200 nt), *L. ferrooxidans *(205 nt), actinobacterium Py-F3 (228 nt), and *At. ferrooxidans *(253 nt).

On two occasions (November 2008 and February 2011) both acid streamers and mine drainage waters were sampled at the same locations within the Dyffryn Adda and bacterial populations compared. Data from the November 2008 samples are shown in Figure 12. One major difference found at both times was that the iron-oxidizing autotroph *Leptospirillum ferrooxidans* accounted for a far greater part of the summated bacterial T-RFs in the mine water (average of 12.5% and 1.4% in 2008 and 2012, respectively) than in the acid streamers (corresponding mean values of 0.7 and 0.6%, and frequently not detected). The actinobacterium isolate Py-F3 showed the opposite trends (acid streamer abundance of 4.6% (2008) and 11.5% (2011) as opposed 0.7% (2008) and 1.5% (2011) in the mine water samples. In contrast to the bacterial communities, the relative abundances of the different archaeal T-RFs in the mine drainage water were found to be similar to those in the acid streamers on the single occasion (November 2008) when this was assessed (Figure 11).

## 3. Discussion

This study has provided unique insights into how a newly-created extremely acidic environment becomes colonized by prokaryotic micro-organisms. The channel, which was excavated to divert subterranean water from the shafts and adits in the underground Mynydd Parys mines into an existing unpolluted stream, became heavily colonized with microbial “streamer” growths within a few months of its construction. These macroscopic growths have continued to occupy most of the volume of the AMD stream over the nine year study period. Analysis of the indigenous microbial populations has shown that, while the same three species of iron-oxidizing bacteria (“*Fv. myxofaciens*”*, At. ferrivorans* (autotrophic species) and isolate Py-F3, which is, as described below, a novel heterotrophic species) have been the dominant members of the streamer communities, there has been a subtle but notable increase in biodiversity during the period of study. Obligately heterotrophic bacteria (e.g., gammaproteobacterium WJ2, *Acidobacterium*, *Acidiphilium* and *Alicyclobacillus* spp.) were detected more regularly by T-RFLP analysis six months after AMD first flowed through the Dyffryn Adda, though non iron-oxidizing heterotrophic acidophiles were always relatively minor, and often more transient, members of the streamer communities. A main reason why the streamer community is thought to be so stable is the near constant physico-chemical nature of the mine water. Its temperature varied by <1 °C (independent of season) over the study period, and its pH and content of ferrous iron (the main energy source in the stream water) and other dissolved solutes have also shown relatively small variations. It is interesting to note that, even though most of the bacteria in the acid streamers are iron-oxidizing acidophiles, only small changes in ferrous iron concentrations were detected between the adit portal and the point at which the Dyffryn Adda joined with the second stream. Water flows continuously from the underground mine, and the stream has never been observed to dry up, even during periods of prolonged lack of precipitation.

Most, if not all, of bacteria and archaea that comprise the acid streamer communities originate from the underground mine water body, the biogeochemistry of which has been described in detail elsewhere [2]. The microbial populations in the underground mine water were analyzed on several occasions during the de-watering of the mine, and bacteria identified included iron-oxidizing acidithiobacilli (*At. ferrivorans* and/or *At. ferrooxidans*), other iron-oxidizers (*L. ferrooxidans* and *Gallionella*-like bacteria (autotrophs) and the heterotrophic acidophile *Fm. acidiphilum*) and non iron-oxidizing heterotrophs (*Acidiphilium, Acidobacterium *and *Acidisphaera* [8]). When the water level within the mine was lowered, large microbial growths that had previously been submerged in the underground water body and which were described by enthusiasts exploring the mine as “drapes” were revealed. Analysis of these “drapes” showed that they were dominated by acidophilic heterotrophic bacteria (*Acidobacteria, Acidisphaera*, gammaproteobacterium WJ2 and species of actinobacteria) in contrast to the Dyffryn Adda acid streamers. The most surprising difference between the subterranean water and “drapes” and the acid streamers in the Dyffryn Adda was that “*Fv. myxofaciens*”, the most abundant bacterium in the latter, was not detected in macroscopic growths or the water body in the underground mine. A similar scenario was reported at a pilot plant used to oxidize and precipitate iron from moderately acidic (pH 4.8) ferruginous ground-water in Nochten, Germany [23]. After a few months of continuous operation, “*Ferrovum*”-like bacteria were found to dominate the microbial communities in the pilot plant, even though other bacteria (*At. ferrooxidans* and *L. ferrooxidans*) had been used as inocula, and *Ferrovum *was not detected in the ground-water.

Autotrophic iron-oxidizing acidophiles have dominated the acid streamers in the Dyffryn Adda from its inception to the present day. An average of 72% of the bacterial community (as assessed by T-RFLP analysis) in the streamers over a nine year period was accounted for by two species, “*Fv. myxofaciens*” and *At. ferrivorans* (Figure 8b). Ferrous iron, continually supplied at ~600 mg/L in the inflowing mine water, represents the major energy source available (excluding sunlight) in the Dyffryn Adda. No reduced sulfur compounds were detected and acidophiles, such as *Acidithiobacillus thiooxidans*, that use only reduced forms of sulfur as electron donors, were also never detected. Dissolved organic carbon concentrations are relatively small, and how much of this is metabolized by acidophilic heterotrophs is unknown. The reasons for “*Fv. myxofaciens*” and *At. ferrivorans* being more successful “streamer” bacteria than other autotrophic iron-oxidizing acidophiles, such as *At. ferrooxidans *and *L. ferrooxidans*, probably relates to (i) their propensity to produce large amounts of extracellular polymeric substances, which causes them to grow (particularly in the case of “*Fv. myxofaciens*”) as visible filaments and flocs *in vitro* [24], and (ii) to both species being psychro-tolerant (growing at 4 °C and above [18]), and therefore well adapted to the constant 11 °C mine water.

The ratio of bacteria to archaeal cells in the acid streamers was not determined in the present study, though analysis of streamers in the Afon Goch at Mynydd Parys [10] and at another acidic mine in north Wales [25] found that archaea accounted for only small fractions of the total microbial populations, at most. The current study indicated that diversity of archaea in the acid streamer growths was much more limited than that of the bacteria. No archaea were isolated, in contrast to bacteria, and all of the clones obtained were very distantly related to known species (though all appeared to be euryarchaeotes), precluding assigning physiological traits to the indigenous archaea.

The microbial composition of the acid streamers in the Dyffryn Adda contrasts greatly with some other streamer/mat growths that have been reported. In some cases, this is readily attributable to the contrasting physico-chemistries of the waters in which they form. For example, the much warmer (35–45 °C) and more acidic (pH 0–2) waters within the Richmond mine at Iron Mountain selects for more acidophilic and thermotolerant species (*Leptospirillum ferriphilum* and “*Ferroplasma acidarmanus*”) while the extremely acidic (pH 0–1) cool (13 °C) sulfur-rich but iron-poor waters in the Frasassi cave select for sulfur-oxidizing bacteria, such as *At. thiooxidans* and *Sulfobacillus* spp. The abandoned Cantareras copper mine in south-west Spain, which has drainage water of similar chemical composition to that at the Dyffryn Adda, provides a more direct contrast [26]. Although the two major bacteria detected in the Dyffryn Adda streamers (“*Fv. myxofaciens*” and *At. ferrivorans*) were also found in the layered streamer/mat communities in a drainage channel at the Cantareras mine, heterotrophic bacteria (e.g. *Acidobacterium* and *Acidiphilium* spp., and novel species of acidophilic sulfate-reducers) were more abundant. This was considered to be due to extensive colonization of the streamer surfaces with acidophilic micro-algae, such as *Chlamydomonas acidophila*, which can provide the organic carbon used by heterotrophic bacteria [27]. Occasional surface growth of *Euglena *may also be the reason for the varying abundance and diversity of heterotrophic acidophiles found in the Dyffryn Adda. Why algal growths are far less extensive here than in the Cantareras mine is not known, but could be related to the lower temperature, regional differences in light intensity, and a generally higher ferric iron content of mine water at the Welsh site.

Based on comparisons of 16S rRNA gene sequences, two of the bacteria isolated from the Dyffryn Adda streamers, Py-H1 and Py-H3, appear to be novel species of *Acidocella* and *Acidiphilium*, respectively, while a third (Py-F3) appears to represent a novel genus. Isolate Py-F3 is an actinobacterium, 100% identical (16S rRNA gene sequence) to a bacterium that was previously also isolated from Dyffryn Adda streamers, KP1 [10]. Isolate Py-F3 appears to have similar physiological characteristics to an obligately acidophilic iron-oxidizing bacterium (CCH7) that was isolated from acid streamer growths within an abandoned pyrite mine [28] but not sequenced. Both Py-F3 and CCH7 grow as long filaments that entwine, forming gelatinous streamer-like growths *in vitro*. Both isolates Py-F3 require organic carbon and oxidize ferrous iron, though in the case of isolate CCH7 it appeared that energy from iron oxidation was not conserved. The nearest classified relative to isolate Py-F3 is *Ferrimicrobium acidiphilum*, which is also an obligately heterotrophic, iron-oxidizing acidophile, but which grows as single rods or short chains, and with a 16S rRNA gene identity of < 93% is clearly a separate genus. The binomial “*Acidithrix ferrooxidans*” is proposed for isolate Py-F3 (denoting its ability for filamentous growth and to oxidize iron) and work on fully characterizing this novel actinobacterium is continuing. Interestingly, another apparently novel actinobacterium (clone PMC25) was detected, but has not yet been isolated, in the streamers. The 16S rRNA gene sequence identities to both *Fm. acidiphilum* (95%) and “*Acidithrix ferrooxidans*” (< 92%) suggest that this is another novel species (or genus) of acidophilic actinobacteria.

## 4. Experimental Section

### 4.1. Site Description and Sampling Regime

Acid mine drainage from the underground Mynydd Parys mines (latitude 53.396784N; longitude-4.350629) following its partial de-watering in 2003, flows first through a buried pipe (*ca*. 50 m long) before entering the newly formed open channel in the upper reaches of the Dyffryn Adda stream, via a screened portal. Some 90 m downstream of the portal, the Dyffryn Adda converges with a relatively unpolluted stream, draining agricultural land, and the conjoined streams flow in a northerly direction for about 1.5 km before entering the Irish Sea. The chemistry and microbiology of the AMD stream at Dyffryn Adda was monitored for over nine years, from October 2003 (three months after the subterranean drainage from Mynydd Parys was diverted into this channel) to August 2012. Mine water chemistry was determined at monthly intervals between October 2003 and November 2007 and less frequently afterwards (only when acid streamers were also analyzed, a total of twelve sampling events).

### 4.2. Physico-Chemical Analyses

On-site measurements of temperature, conductivity, pH, dissolved oxygen (DO) and redox potentials (*E*_h_; corrected to be relative to a standard hydrogen electrode reference electrode) were carried out using a YSI 556 MPS multi-meter (YSI instruments, Yellow Springs, Ohio). For laboratory analyses, water samples were filtered through 0.2 μm cellulose nitrate membrane filters into sterile sample tubes, with a sub-set of each sample acidified (by adding two drops of concentrated nitric acid) for subsequent analysis of transition metals (using ion chromatography [29]). Concentrations of sulfate were determined using a turbidometric technique [30] or by ion chromatography, ferrous iron using the ferrozine assay [31], and dissolved organic carbon (DOC) using a LABTOC DOC analyzer (Pollution & Process Monitoring Ltd., UK).

### 4.3. Molecular Analysis of Acid Streamer Growths

Samples of acid streamers (50 cm^3^) were removed from the stretch of the Dyffryn Adda between the upper portal and the point where it joined the second stream, and put into sterile Falcon tubes. Samples were maintained at 4 °C and processed within 24 hours of collection.

Terminal restriction enzyme fragment length polymorphism (T-RFLP) was used routinely to determine the compositions of the microbial communities of acid streamer samples. This is a semi-quantitative, PCR-based technique that has been used successfully to assess similar growths at other mine sites (e.g., [25]). In brief, DNA was extracted from the acid streamer samples (0.1 g) using the MO-BIO Ultraclean Soil DNA Isolation kit (MO-BIO Laboratories Inc. USA) according to the manufacturer's instructions. Then 16S rRNA gene amplifications were performed in triplicate and products combined to avoid PCR bias [32] using Cy5-labelled fluorescent forward primers. Amplification primers for bacterial 16S rRNA genes were the forward primer 27F (5'-AGAGTTTGATCCTGGCTCAG-3'), and the reverse amplification primers were either 1387R (5'-GGGCGGWGTGTACAAGGC-3'), 1492R (5'–TACGGYTACCTTGTTACGACT-3') or 536R (5' -CAGCSGCCGCGGTAAWC-3'). For Archaeal 16S rRNA gene amplifications the primers used were 20F (5'–TCCGGTTGATCCYGCCRG-3') and 915R (5'–GTGCTCCCCCGCCAATTCCT–3'). Bacterial PCR conditions were: 95 °C (5 min), followed by 30 cycles at 95 °C (30 s), 55 °C (30 s) and 72 °C (1.5 min), and a final extension at 72 °C (10 min). The conditions for archaeal PCR amplifications were 95 °C (5 min), followed by 30 cycles at 95 °C (30 s), 62 °C (30 s) and 72 °C (1.0 min), and a final extension at 72 °C (10 min).

The resulting PCR fragments were purified using SureClean (Bioline Ltd., UK), and digested separately with up to three restriction endonucleases; HaeIII, CfoI or AluI (Promega, UK) to differentiate the 16S rRNA gene fragments by producing digestion products of differing terminally labeled restriction fragment lengths (T-RFs). The digested products were analyzed by capillary electrophoresis (CEQ8000 genetic analysis system; Beckman-Coulter, UK) and sized by their mobility in comparison with fluorescently labeled size standards. Total relative abundances (%) of the individual T-RFs were calculated from peak areas which are directly related to the fluorescent peak intensity, e.g., large peak intensity indicates a greater total abundance in the environmental samples. Identification of the major micro-organisms represented by individual T-RFs was facilitated by comparison of the latter against a database maintained at the authors' laboratory, which was compiled from extensive T-RF data obtained from acidophilic bacterial and archaeal isolate and clone sequences. In cases where T-RFs from all three enzyme digests corresponded to a known micro-organism, identification was classed as “firm”, whereas if only two of the three digests corresponded to a known micro-organism, identification was regarded as “tentative”.

### 4.4. Molecular Analysis of Planktonic-Phase Bacteria

On two occasions (November 2008 and February 2011) the bacteria present in the AMD flowing through the Dyffryn Adda were also analyzed using T-RFLP of extracted DNA. Approximately, 500 mL of mine water was filtered (sterile 0.22 μm cellulose nitrate filters) at the adit site and the filters were stored at −20 °C until DNA extraction. DNA extraction used the same kit and protocol as stated previously except whole filters were aseptically cut into pieces and placed into the bead beating tube. The extracted DNA was used as template for bacterial and archaeal 16S rRNA T-RFLP PCR using the fluorophore-labeled primers and conditions as stated above.

### 4.5. Clone Library Construction

For the construction of a clone library of 16S rRNA genes, unlabeled PCR fragments were generated as described above, purified and ligated into the pGem-T-Easy vector system (Promega, UK) according to the manufacturer's instructions. Insert-vector ligation mixes (3:1 ratio) were used to transform *Escherichia coli *DH5α [33] and cells plated on selective media. The 16S rRNA gene inserts in the clones were amplified by PCR, differentiated via restriction enzyme fragment length polymorphism (RFLP), and visualized using agarose gel electrophoresis. Plasmid inserts that generated distinct RFLP patterns were further screened using T-RFLP to ascertain whether these corresponded to unknown T-RFs in streamer profiles. These were isolated with Strataprep plasmid mini-prep kits (Agilent technologies, USA) and sequenced (Macrogen, Inc., Korea). Chromatogram files were visualized using Chromas Lite, sequences edited to generate a contiguous gene sequence for each isolate, and then these were aligned using the BlastN on-line software (NCBI). Gene sequences were compared to those contained in the Genbank database.

### 4.6. Cultivation Analysis of Acid Streamer Bacteria

Small fragments (*ca*. 0.5 cm^3^) of acid streamer samples were removed from the bulk samples using sterile tweezers, placed in 1 ml of sterile acidic (pH 2.5) water, and bacterial cells were dislodged by vortexing for 20 s. The streamer remnants were removed by gentle centrifugation and the cell suspensions streak-inoculated onto a range of solid overlay and non-overlay media formulated to promote the growth of autotrophic and heterotrophic acidophiles [34]. Plates were incubated for 3–4 weeks at 30 °C and inspected on a regular basis. Preliminary identification of isolates that grew on the different media was based on their colony morphologies (e.g. ferric iron-stained colonies that grew on ferrous iron media were identified as acidophilic iron-oxidizers) and their cell morphologies (e.g. highly motile curved rods that formed ferric iron-stained colonies were identified as *Leptospirillum* spp.). To confirm their identities, bacterial isolates were purified by repeated single colony isolation on solid media, and PCR amplification of 16S rRNA genes carried out using either single colonies (from plates) or pellets (from liquid cultures). These were sequenced by Macrogen using the 27F and 1387R primer pair.

## 5. Conclusions

A long-term study of microbial communities in a new drainage channel receiving acidic (pH 2.5) water from an abandoned copper mine found that acid streamer growths rapidly colonized the drain channel and displayed changes in community profiles with time. Three iron-oxidizing bacteria dominated the streamer communities, two of which (*Acidithiobacillus ferrivorans* and “*Ferrovum myxofaciens*”) were obligate chemolithotrophs and the third (a novel genus/species with the proposed name “*Acidithrix ferrooxidans*”) a heterotrophic acidophile. With time, the biodiversity of the acid streamers increased, though other bacteria that were identified were always present in relatively low abundance. Archaea were only detected in the streamer growths some time after the drainage stream was established, and showed limited diversity. This study provides rare insights into how microbial communities colonize and evolve in a newly-created extreme environment.
